# Biochar Improves Soil-Tomato Plant, Tomato Production, and Economic Benefits under Reduced Nitrogen Application in Northwestern China

**DOI:** 10.3390/plants10040759

**Published:** 2021-04-13

**Authors:** Lili Guo, Huiwen Yu, Mourad Kharbach, Wenqian Zhang, Jingwei Wang, Wenquan Niu

**Affiliations:** 1 College of Water Resources and Architectural Engineering, Northwest A&F University, Weihui Road 23, Yangling 712100, China; Mia@nwsuaf.edu.cn (L.G.); wqzhang@nwafu.edu.cn (W.Z.); 2 Department of Plant and Environmental Science, Faculty of Science, University of Copenhagen, Højbakkegaard Alle 13, DK-2630 Taastrup, Denmark; 3 Key Laboratory of Agricultural Soil and Water Engineering in Arid and Semiarid Areas, Ministry of Education, Northwest A&F University, Yangling 712100, China; 4 Department of Food Science, Faculty of Science, University of Copenhagen, Rolighedsvej 26, DK-1958 Frederiksberg C, Denmark; huiwen.yu@food.ku.dk; 5 Research Unit of Mathematical Sciences, University of Oulu, FI-90014 Oulu, Finland; mourad.kharbach@hotmail.fr; 6 College of Resources and Environment, Shanxi University of Finance and Economics, Taiyuan 030000, China; wjw@sxufe.edu.cn; 7 Institute of Soil and Water Conservation, Northwest A&F University, Yangling 712100, China; 8 Institute of Soil and Water Conservation, CAS &MWR, Yangling 712100, China

**Keywords:** biochar, N fertilizer reduction, microenvironment, tomato growth, optimal biochar‒N combination

## Abstract

The tomato is an important economic crop that is a main ingredient of some prepared food as well as a focus of the agricultural industry. Optimizing nitrogen (N) fertilizers is essential for sustainable agricultural development, while the excessive use of N fertilizers leads to environmental and food production problems. As a soil amendment, biochar has been widely used to improve soil quality and crop yield. However, little information is available on the effects of biochar and N fertilizer reduction on tomato plant, soil characteristics in tomato cultivation and tomato production. In this study, a greenhouse experiment was carried out in Yangling, Shaanxi province, China, including four biochar levels (0, 30, 50, and 70 t ha^−1^) under drip irrigation and four N application rates (170, 190, 210, and 250 kg ha^−1^). The results showed that adding too much biochar (e.g., 70 t ha^−1^) and reducing N fertilizer too far (e.g., by 32%) will not lead to satisfactory results in terms of tomato growth, tomato yield and quality, and economic benefits. Biochar addition could significantly enhance microbial abundance, enzyme activity, and tomato growth compared with non‒biochar treatments when reducing the amount of applied N fertilizer by 16% or 24% (N2 and N3). From the perspectives of tomato yield, tomato quality (sugar‒acid ratio and vitamin C (VC) content), and economic benefits, optimal application rate of biochar and N fertilizer based on the silty clay loam soil of northwest China under drip irrigation is proposed, respectively. The proposal is based on both multidimensional nonlinear regression models and a comparison with experimental treatments. For example, biochar addition at 50 t ha^−1^ and reducing N fertilizer by 24% achieved the greatest tomato yield. Compared with non-biochar treatment under the corresponding N fertilizer level, soil enzyme activity (urease, phosphatase, and catalase), microbial abundance (bacteria, fungi, and actinomycetes), leaf gas exchange parameters (gs, Pn, and Tr), and biomass increased on average by 88.76%, 7.49%, 43.23%, and 39.67%, respectively. Based on a comprehensive consideration of tomato yield, VC content, sugar‒acid ratio, and economic benefits, 35 t ha^−1^ biochar and 200 kg ha^−1^ N fertilizer is the recommended combination of biochar and nitrogen fertilizer for local farmers.

## 1. Introduction

Nitrogen (N) fertilizer accounts for about 60% of total fertilizer consumption in China, and the annual consumption of N fertilizer accounts for more than 35% of the world’s total consumption, which is increasing year on year [1]. Excessive use of N fertilizer is a serious problem that leads to low fertilizer utilization and a decline in crop yields [2]. Therefore, it is urgent to explore effective fertilization measures to improve soil fertility and crop productivity. Many studies have shown that the combined application of biochar and N fertilizer is beneficial to different crops’ growth and yield, e.g., it could increase the productivity of rapeseed and sweet potato planting in dryland red soil [3], promote maize’s absorption of N, and increase the maize yield [4]. However, Majeed et al. [5] reported that the type and dosage of biochar and the dosage of N fertilizer were all effective factors affecting soil fertility and maize growth. In addition, the highly porous structure of biochar provides a habitat for microorganisms to settle down, so that they can grow better in the soil environment [6,7]. Some studies have reported that the amount of biochar also affects changes in soil microorganisms [8]. The tomato is rich in nutrients and is one of the most widely cultivated vegetables [9]. As an important economic crop, it is the main ingredient in some prepared food as well as a focus of the agricultural industry [10]. However, there are few studies on the effects of the combined application of biochar and N fertilizer on tomato soil-plant system, tomato production and fruit quality. The influence mechanism and applied strategy of biochar and N fertilizer for tomato plants under drip irrigation are still unclear. 

Therefore, it is necessary to study the reasonable combined dosage of biochar and N fertilizer for improving tomato plant growth, tomato production, and fruit quality [11]. The objective of this study was to investigate the effect of the combination of biochar and N fertilization on tomato soil-plant system, fruit quality and tomato production by revealing the influence mechanism and the response of the soil microenvironment to C‒N. The economic benefits of tomatoes under different applied biochar and N fertilizer combination are also calculated and compared. After calculating the multidimensional nonlinear regression models [12], the optimal application of biochar and N fertilizer is recommended to achieve high quality, high yield and high economic benefits in the test area. 

## 2. Methods and Materials 

### 2.1. Experimental Site and Materials

The experiment was conducted in a greenhouse from 6 April 2018 to 1 August 2018 in Yangling City, Shaanxi Province, China (520 m altitude, 34°17′ N and 108°02′ E). The area has a semi-humid continental monsoon climate with an average annual temperature of about 16.1 °C. The annual mean sunshine duration is 2165 h and the mean annual frost-free period is more than 210 days. The greenhouse was 108 m in length and 8 m in width. The area of each plot was 6.5 m in length and 3.2 m in width. The soil texture was classified as a silty clay loam according to the USDA classification system [13], with 25.4% gravel (2–0.02 mm), 44.1% silt (0.02–0.002 mm), and 30.5% clay (<0.002 mm). The soil had a field capacity of 27.98%, a pH of 7.35, a bulk density of 1.35 g cm^−3^, and a soil porosity of 49.01%. The organic matter content was 16.48 g kg^−1^, total N was 0.96 g kg^−1^, total P was 0.87 g kg^−1^, and total K was 10.4 g kg^−1^.

The pyrolysis temperature of biochar was 450 °C, and the feedstock was the trunk and branches of discarded fruit trees (Shaanxi Yixin Bioenergy Technology Development Co., Ltd., Yangling, China). The biochar had a specific surface area of 87.1 m^2^ g^−1^, and a pH of 10.51. The content of carbon was 72.38%, total N was 0.98 g kg^−1^, N-NO_3_ was 0.59 mg kg^−1^, and N-NH_4_ was 1.67 mg kg^−1^. The tomato variety used was “Dorui Star” (Seedling Breeding Center of Yangling Demonstration Area, Yangling, China). Drip irrigation pipes (Shaanxi Huawei Agricultural Science and Technology Development Co., Ltd., Yangling, China) were laid on the ground, and the length was the same as the ridge length (5.5 m). The N fertilizer, phosphate (P) fertilizer, and potassium (K) fertilizer used in the study were urea (N = 46% by weight), biological phosphorus (P_2_O_5_, P = 16% by weight) [14], and potassium sulfate (K_2_O, K = 51% by weight). A total of 150 kg P_2_O_5_ ha^−1^ and 200 kg K_2_O ha^−1^ were applied as basal fertilizer.

### 2.2. Experimental Setup

Similar to the experimental design of [1,3,12], our experiment consisted of four biochar levels under drip irrigation (C0, 0 t ha^−1^; C30, 30 t ha^−1^; C50, 50 t ha^−1^; C70, 70 t ha^−1^), and four control N application rates (N1, 170 kg ha^−1^; N2, 190 kg ha^−1^; N3, 210 kg ha^−1^; N4, 250 kg ha^−1^). N4 is the normal amount of N fertilizer used by local farmers. N1, N2, and N3 were reduced by 32%, 24%, and 16%, respectively, compared with N4 application. A 4 × 4 complete combination was used, which led to a total of 16 treatments. Each treatment occupied a block in the greenhouse. One block was divided into three small plots (replicates), and 30 plants were transplanted to each plot in double rows with a row distance of 40 cm and plant spacing of 45 cm. Before the experiment, the biochar (passed through a 4-mm sieve) was applied to the 0‒30 cm soil layer by ploughing with a rotary tiller, and then N fertilizer and basal fertilizers were applied. 

### 2.3. Measurements

#### 2.3.1. Soil Enzyme Activity and Microbial Abundance

In this study, soil samples were collected at a depth of 0 to 20 cm at the tomato maturation stage (90 days after planting) for soil enzymatic activity and soil microbial abundance measurements [14]. Each treatment had three replicates. 

Urease enzyme in the soil is known to have the ability to catalyze urea-N hydrolysis to NH_3_ [15]. Five grams of soil, 10 mL of 10% urea solution, and 20 mL of citrate buffer (pH = 6.7) were added to a 50 mL flask and incubated at 37 °C for 24 h [16]. The urease activity was then determined by spectrophotometry at 578 nm. Phosphatase enzymes are of crucial importance in the release of bioavailable inorganic P from organic P in the soil [17]. One gram of soil sample, 0.2 mL of toluene, 4 mL modified universal buffer (pH = 8.5), and 1 mL of p-nitrophenyl phosphate solution were added to 50-mL glass vials and incubated at 37 °C for 1 h [18]. The p-nitrophenol released during enzymatic hydrolysis was measured by a spectrophotometer at 400 nm, which was the quantification of phosphatase activity. Catalase can catalyze the breakdown of hydrogen peroxide into oxygen and water and regulate hydrogen peroxide metabolism [19]. The catalase activity measured by the titration method was expressed as the milliliters of KMnO_4_ solution consumed by each gram of soil (in units of mg L^−1^) [14]. Soil microbial abundance was deemed to be an important biological indicator used to evaluate soil fertility [20]. The abundance of bacteria, fungi, and actinomycetes in the soil was determined by the dilution plate method in beef extract-peptone, Mardin’s media, and the improved Gause’s No. 1 growth media, respectively. The media plates were incubated at 37 °C and 25 °C, and the number of colonies was counted after approximately 3‒5 days [21].

#### 2.3.2. Tomato Leaf Gas Exchange Parameters

Five plants were selected in each plot, which means that 15 tomato plants were measured for each treatment. After this, five healthy leaves with sufficient light exposure and consistent leaf position were semi-randomly selected from each plant. The photosynthetic rate (Pn), stomatal conductance (gs), and transpiration rate (Tr) of the leaves were measured by the LI-6400 photosynthesis measurement system (LI-COR, Lincoln, NE, USA) [22]. The measuring time was 10:00‒12:00. The leaves were measured every 20 days after planting a total of five times, and the final result was the average of the five measurements.

#### 2.3.3. Tomato Growth and Biomass 

In the tomato maturity period (80 days after planting), a dynamic change in tomato plants growth was observed. The plant height was determined from the soil line to the tip of the main stem using a steel ruler, and the stem diameter of tomatoes was measured weekly by a digital Vernier caliper at the thickest place of the main stem, which was 10 cm above the ground [23]. After harvesting the plants (110 days after planting), we carefully removed the soil mass and picked up the residual root system. The stems, leaves, and fruits were separated, and the roots were rinsed with water. The roots, stems, leaves, and fruits were put into an oven at 105 °C for 30 min, then were totally dried at 75 °C and weighed [24]. 

#### 2.3.4. Tomato Quality, Yield, and Partial-Factor Productivity of N Fertilizer

In the tomato harvest period (81‒110 days after planting), the first layer to the fourth layer fruit of the aforementioned 15 tomato plants for each treatment were measured. The total yield of each plant was the cumulative output of the four layers of fruit. Then, the second layer of these tomato fruits was selected for quality measurement [25]. VC, soluble sugar, and organic acid were determined by ultraviolet spectrophotometer (Thermo Fisher, Waltham, MA, USA) [26]. The ratio of sugar to acid (sugar‒acid ratio) is the ratio of soluble sugar and organic acid. VC and the sugar‒acid ratio are used to measure the tomato quality in this study. VC is a nutritional indicator in tomatoes [27] and the sugar‒acid ratio is an indicator of taste [28].

The partial-factor productivity of applied N (PFP, kg kg^−1^) is a useful measure of nutrient-use efficiency [29] and is calculated as follows:

PFP = TY/TN(1)

where TY is the tomato yield (kg ha^−1^) and TN is the total input of N fertilizer (kg ha^−1^).

#### 2.3.5. The Yield, Quality of Tomatoes, and Economic Benefit Model

Multidimensional nonlinear regression models [3] are usually used to study the relationship between multiple variables and single dependent variables. This study uses a ternary nonlinear regression model to study the relationship between tomato yield (quality or economic benefit), biochar, and N fertilizer application. 

The basic formula of the model is:

z = z0 + ax + by + cxy + dx^2^ + ey^2^ (2)

where z is the dependent variable, z0 is a constant, x and y are independent variables, xy is an interactivity term, and x^2^ and y^2^ are the square terms of the independent variables.

For fitting the economic benefit model, both variable costs (e.g., biochar cost) and fixed costs (e.g., pipe system cost) were taken into account. The price of biochar and N fertilizer is 2 yuan kg^−1^ and 1.7 yuan kg^−1^, respectively, and the tomatoes were sold at a price of 5.6 yuan kg^−1^. More details of costs and benefits for all the treatments in single greenhouse are shown in Table A1.

### 2.4. Statistical Analysis

All data were expressed as treatment mean ± standard error. Data were prepared using Microsoft Excel (Microsoft Corporation, Redmond, WA, USA). The statistical analysis was performed using SPSS version 23.0 software (SPSS Inc., Chicago, IL, USA). The principal component analysis (PCA) of all indicators in this experiment was conducted with MATLAB R2018a software (MathWorks, Inc., Natick, MA, USA). The data were subjected to two-way analysis of variance (ANOVA) for the independent variables: biochar ©, N fertilizer (N), and their interactions (C × N). Duncan’s range at the 5% confidence level was used to test for significant differences. All graphs were prepared using MATLAB R2018a software. 

## 3. Results

### 3.1. PCA Analysis of Soil‒Plant Parameters as Affected by the Treatments

**Figure 1:**
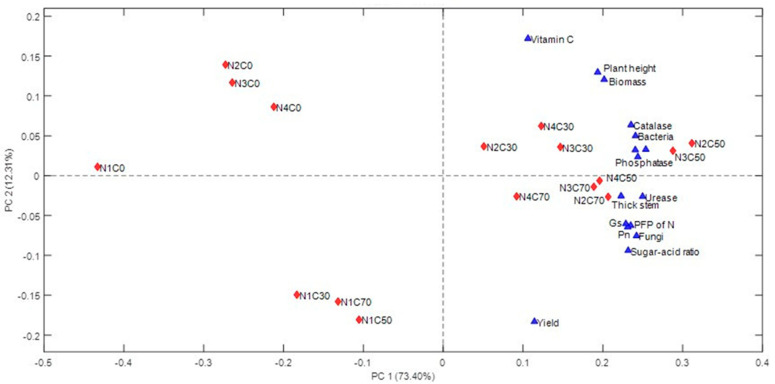
Principal component analysis (PCA) of all the measured soil‒plant parameters.

The PCA analysis, which was done based on the measured soil‒plant parameters, revealed that the treatments were separated into distinct clusters (Figure 1). The treatments were divided into four groups, including a first group without any biochar addition (N1C0, N2C0, N3C0, and N4C0), a second group with maximum N fertilizer reduction (N1C30, N1C50, and N1C70), a third group with a combination of N2, N3, N4 and C30, C50 (N2C50, N3C50, N4C50, N2C30, N3C30, and N4C30), and a fourth group with the maximum biochar addition (N2C70, N3C70, and N4C70). The PC1 explained 73.40% of the variation, whereas PC2 explained only 12.31% of the variation. All of the soil‒plant parameters were on the right side of the plot along the PC1 direction, which means there is an opposite relationship between the left and right treatments in terms of these soil‒plant parameters. That is to say, there is a significant soil‒plant parameter difference between biochar addition treatments and non-biochar treatment, as well as between maximum N fertilizer reduction treatments and other N fertilizer reduction treatments. 

### 3.2. Soil Microbial Abundance and Enzyme Activity

The two-way ANOVA results in Table 1 show that the activity of urease, phosphatase, and catalase, and the quantity of bacteria, fungi, and actinomycetes were all significantly affected by N, C, and their interaction, N × C. The Duncan’s test (Table 2) showed that the activity of urease, phosphatase, and catalase, and the quantity of bacteria, fungi, and actinomycetes of the biochar addition treatments significantly increased compared with non-biochar treatment when ignoring the level of N fertilizer. They first increased and then decreased as the amount of added biochar increased, and they reached the maximum in either the C30 or C50 treatment. The activity of urease, phosphatase, and catalase, and the quantity of bacteria, fungi, and actinomycetes of the N1 treatments decreased significantly compared with the other N treatments. 

Concerning all the treatments with biochar and N, the highest urease activity was seen for N4C30, which was 33.94% greater than the urease activity of N4C0; the largest quantity of fungi was seen for N3C30, which was 89.44% greater than the quantity of fungi in N3C0; the largest quantity of bacteria and actinomycetes, and the highest phosphatase activity and catalase activity, was seen for N2C50—89.44%, 87.87%, 55.51%, and 179.63% greater, respectively, than that of N2C0. For the C0 treatment group (no biochar addition), the values of soil enzyme activity and microbial abundance decreased as the amount of N fertilizer application decreased. For the C30 treatment group, there was no significant difference in enzyme activity and microbial abundance between the N3C30 and N4C30 treatments, which means that adding 30 t ha^−1^ biochar can result in 16% N fertilizer reduction without any significant loss in soil microorganism. For the C50 treatment group, there was no significant difference in terms of urease activity, phosphatase activity, and number of actinomycetes among the N2C50, N3C50, and N4C50 treatments. In particular, the catalase activity and the number of fungi and bacteria in the N2C50 treatment were significantly greater than in the N4C50 treatment. A similar pattern can be observed in the C70 treatment group. Thus, more N fertilizer (from 210 kg h^−1^ to 190 kg ha^−1^) can be reduced as the added biochar increased without a significant loss in the quantity of soil microorganisms. However, it is worth mentioning that N1 combined with the C0, C30, C50, and C70 treatments resulted in a significant loss in soil microorganisms.

### 3.3. Leaf Gas Exchange Parameters

The two-way ANOVA results showed that the leaf gas exchange parameters Pn, Tr, and Gs were significantly affected by N, C, and their interaction, N × C (Table 3). In Table 4, Duncan’s test shows that the Pn, Tr, and Gs of biochar treatments significantly increased compared with non-biochar treatments, and reached a maximum in either the C50 or C70 treatment. All the N1 treatments had a significant loss in Pn, gs, and Tr compared with the other N treatments. 

Regarding the interaction of C and N, the largest Pn occurred for N3C50, which increased by 48.93% compared with N3C0; N2C50 achieved the largest gs, which increased by 58.97% compared with N2C0; N3C70 achieved the largest Tr, which increased by 49.62% compared with N3C0. For the C30 treatment group, Tr and Pn in the N3C30 treatment were greater than in the N4C30 treatment. For the C50 and C70 treatment groups, no significant differences in Pn, gs, and Tr were found among the N2C50, N2C70, N4C50, and N4C70 treatments, even though the Pn and Tr in the N3C50 treatment were significantly greater than in the N4C50 treatment. It is concluded that N fertilizer application with 210 kg ha^−1^ (N3) is the optimal level for each C treatment (C30, C50, and C70) from the perspective of leaf gas exchange parameters.

### 3.4. Tomato Growth and Biomass

The two-way ANOVA results show that plant height, stem thickness, and biomass were significantly affected by N, C, and their interaction, N × C (Table 3). The Duncan’s test in Table 4 shows that plant height, stem thickness, and biomass in the biochar treatments is significantly greater than in non-biochar treatments. Biochar addition caused these parameters to first increase and then decrease as the amount of added biochar increased; maximum gains were always obtained when the biochar addition was 50 t ha^−1^. The plant height, stem thickness, and biomass of the N1 treatments significantly decreased compared with other N treatments. Concerning the interaction of C and N, N3C50 treatment achieved the highest plant height—an increase of 16.54% compared with N3C0; the N2C50 treatment had the largest amount of biomass—an increase by 39.67% compared with N2C0. For the C0 treatment group, plant height, stem thickness, and biomass decreased as the amount of N fertilizer application decreased. For the C30 treatment group, the amount of biomass in the N3C30 treatment was significantly greater than that in the N4C30 treatment, and there was a significant difference in plant height between N3C30 and N4C30. For the C50 treatment group, there was no significant difference in either stem thickness or the amount of biomass between the N2 and N4 treatments, while the plant height in the N2 treatment was significantly greater than that in the N4 treatment. A similar pattern can also be seen in the C70 treatment group. Hence, a higher amount of N fertilizer reduction is possible (from 16 to 24% reduction) without a loss in tomato growth and biomass as the biochar addition increases from 0 t ha^−1^ to 50 t ha^−1^.

### 3.5. Tomato Quality, Yield of Tomato, and Partial-Factor Productivity for N

The two-way ANOVA results in Table 5 show that the sugar‒acid ratio was significantly affected by N and C. VC, tomato yield, and PFP of N were significantly affected by N, C, and their interaction, N × C. In Table 6, the Duncan’s test shows that VC, tomato yield, and PFP of N in biochar treatment are significantly greater than those in non-biochar treatment. The gains of biochar addition in these parameters first increase and then decrease as more biochar is added, and the maximum gains were obtained when the biochar addition was 50 t ha^−1^. However, the biochar addition of 70 t ha^−1^ significantly reduced the sugar‒acid ratio. The PFP of N decreased as the amount of applied N fertilizer increased. Tomato yield, VC, and the sugar‒acid ratio first increased and then decreased with the increase in N fertilizer. Regarding the interaction of C and N, the largest VC was seen for N3C30 with an increase of 29.36% compared with N3C0 and the largest sugar‒acid ratio was seen for N2C30 with an increase of 3.43% compared with N2C0. The largest tomato yield was seen for N2C50, which increased by 54.79% compared with N2C0, and the largest PFP of N was seen for N1C50, which increased by 59.13% compared with N1C0.

For the C30 treatment group, yield and VC in the N3C30 treatment were greater than in the N4C30 treatment. For the C50 treatment group, yield and VC in the N2C50 and N3C50 treatments were significantly greater than in the N4C50 treatment. A similar pattern was seen for the C70 treatment group.

### 3.6. The Relationship between VC and Sugar–Acid Ratio and Application Rates of Biochar‒N Fertilizer

To study the relationship between yield and quality of tomato and application rates of biochar and N fertilizer, a multidimensional nonlinear regression model was built, in which biochar and N fertilizer were defined as independent variables, while VC and the sugar‒acid ratio were defined as dependent variables. The multidimensional nonlinear regression model is shown below:

(3) $$Y_{1}\;=\;-56.9081\;+\;0.077C\;+\;0.5786N\;-\;0.0001\mathrm{CN}\;-\;0{.0008C}^{2}\;-\;0{.0013N}^{2}\;\left(R^{2}=0.9504\right)$$

(4) $$Y_{2}\;=\;-81.6786\;+\;0.0496C\;+\;0.8537N\;-\;0.0001\mathrm{CN}\;-\;0{.0005C}^{2}\;-\;0{.0020N}^{2}\;\left(R^{2}=0.8396\right)$$

**Figure 2:**
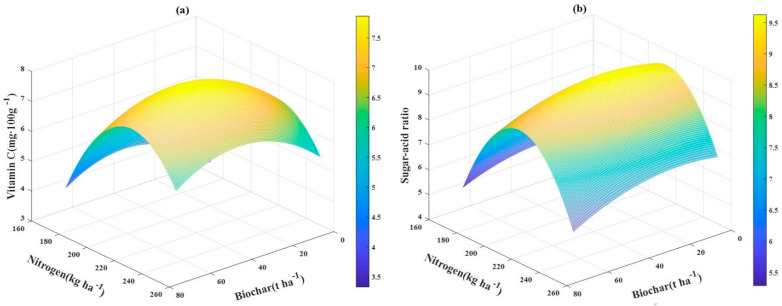
The relationship between VC (**a**) and sugar‒acid ratio (**b**) and biochar C‒fertilizer N application rates as a 3D color maps.

In these equations, Y_1_ denotes the VC, in mg·100 g^−1^; Y_2_ denotes the sugar‒acid ratio; C denotes the biochar application rate, on t ha^−1^; N denotes the N fertilizer application rate, in kg ha^−1^; CN denotes the interactive term between N fertilizer and biochar, and C^2^ and N^2^ are the squared terms of the independent variables. A 3D color map based on the model is shown in Figure 2.

After calculating the multidimension regression model, the maximum VC of tomato was 8.41 mg·100 g^−1^, which could be achieved with a combined application of 34.3 t ha^−1^ biochar and 221.2 kg ha^−1^ N fertilizer, and the maximum sugar‒acid ratio of the tomatoes was 9.82, which could be achieved with a combined application of 28.3 t ha^−1^ biochar and 212.7 kg ha^−1^ N fertilizer. However, compared with the above treatments, achieving the maximum VC and sugar‒acid ratio, N3C30 (VC is 7.96) reduced by 5.10% the N fertilizer with a very small VC loss, and N2C30 (sugar‒acid ratio: 9.59) reduced by 11.90% the N fertilizer with a very small sugar‒acid ratio loss. Thus, the application of 30 t ha^−1^ biochar and 210 kg ha^−1^ N fertilizer was recommended as the best combination for VC in tomatoes, and the application of 30 t ha^−1^ biochar and 190 kg ha^−1^ N fertilizer was the best combination for the sugar‒acid ratio of tomatoes.

### 3.7. The Relationship between Tomato Yield and Economic Benefit and Application Rates of Biochar‒N Fertilizer

To study the relationship between tomato yield and the economic benefits and application rates of biochar and N fertilizer, a multidimensional nonlinear regression model was built in which biochar and N fertilizer were defined as independent variables and tomato yield and economic benefit were defined as the dependent variables. The multidimensional nonlinear regression model is shown below:

(5) $$Y_{3}\;=\;-83.5527\;+\;1.6639C\;+\;1.2574N\;-\;0.0024\mathrm{CN}\;-\;0{.0114C}^{2}\;-\;0{.0025N}^{2}\;\left(R^{2}=0.991\right)$$

(6) $$Y_{4}\;=\;-31.0006\;+\;0.4849C\;+\;0.4437N\;-\;0.0009\mathrm{CN}\;-\;0{.0043C}^{2}\;-\;0{.0009N}^{2}\;\left(R^{2}=0.976\right)$$

**Figure 3:**
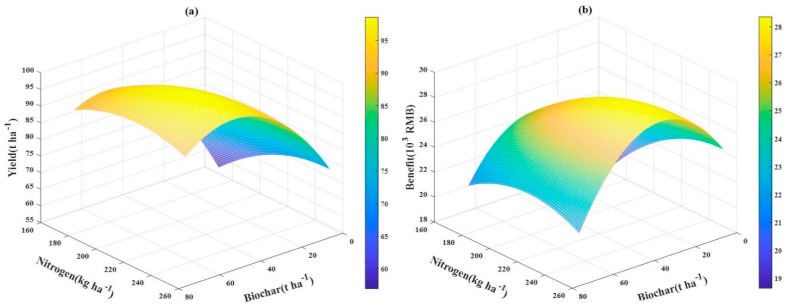
The relationship between tomato yield (**a**) and economic benefit (**b**) and biochar C-fertilizer N application rates in a 3D color map.

Y_3_ denotes the yield, in t ha^−1^, Y_4_ is the economic benefit, 10^3^ CNY; C is the biochar application rate, in t ha^−1^; N is the N fertilizer application rate, in kg ha^−1^; CN is the interactive term between N fertilizer and biochar, and C^2^ and N^2^ are the squared terms of the independent variables. The 3D color map surface based on the model is shown in Figure 3. After computing the multidimensional regression model, the maximum yield of tomato was 100.5 t ha^−1^, which could be achieved with a combined application of 48.9 t ha^−1^ biochar and 227.9 kg ha^−1^ N fertilizer. The maximum economic benefit was 27.93 × 10^3^ CNY, which could be achieved with a combined application of biochar and N fertilizer of 32.3 t ha^−1^ and 230.4 kg ha^−1^. However, compared with the above treatments, for achieving the highest yield and benefit, N2C50 (yield: 96.4 t ha^−1^) will reduce the N fertilizer by 19.95% without a significant loss in yield, while N3C30 (with a benefit of 27.92 × 10^3^ CNY) would reduce the N fertilizer by 8.85% with very little benefit loss. Thus, the application of 50 t ha^−1^ biochar and 190 kg ha^−1^ N fertilizer was recommended as the best combination for tomato yield, while the application of 30 t ha^−1^ biochar and 210 kg ha^−1^ N fertilizer was the best combination in terms of the economic benefit.

## 4. Discussion

In recent years, biochar has been widely used to improve soil ecosystems, optimize agricultural systems, and alleviate environmental problems [30,31]. In this study, it is illustrated in Table 2 that biochar can improve the hydrolysis reactions of urea and the oxidizing ability of soil microorganisms by increasing the activity of urease and catalase. The biochar addition increases the P available for plant growth due to increased phosphatase activity, and improves soil nutrients by increasing the abundance of soil microbial (fungi, bacteria, and actinomycetes). This is consistent with the results reported by Li et al. [32], that the application of biochar as a soil amendment to the soil can improve soil biological characteristics by enhancing soil microbial functional activity and changing the community structure. Biochar has been confirmed to have the ability to change the water content and pH of the soil [33]; this may also be why biochar affects soil enzyme activity and the microbial abundance. Meanwhile, all soil‒plant parameters were on the right side of the plot along the PC1 direction (Figure 1), indicating that the effects of almost all the parameters are positively correlated. Thus, it is concluded that biochar improves the soil microenvironment by promoting soil enzyme activity and microbial abundance (Table 2), which leads to the improvement of tomato leaf gas exchange (Pn, gs, and Tr), plant height, stem thickness, and biomass accumulation (Table 4).

There are a few studies on the effect of biochar on tomato plant growth, production, and quality, and some results are inconsistent. Similar to the results of this study, Guo et al. [33] showed that biochar application improved plant leaf gas exchange under deficit irrigation, as exemplified by the higher Pn, gs, and Tr. Akhtar et al. [34] argued that Pn was affected insignificantly by biochar treatments, even though gs was significantly increased under biochar addition treatments. Moreover, adding biochar is deemed to increase VC content, tomato sugar‒acid ratio, and yield in this study. Agbna et al. [35] showed that biochar could enhance the growth, physiology, and yield of tomatoes compared with non-biochar treatments; this is in agreement with our research. However, Akhtar et al. [34] showed that biochar amendment increased tomato yield, but its impact on VC was not significant. Petruccelli et al. [36] showed that adding biochar did not lead to a significant improvement in tomato fruit size and weight parameters, or sugar content. The reason for this inconsistency may be that different test areas, different soils, and types of biochar will affect the research results. For example, it has been reported that biochar pyrolyzed at different temperatures, differentially affects tomato growth and fruit quality [37]. The results of this research will be beneficial for addressing such inconsistency to some extent.

Furthermore, our study found a significant interaction between biochar and N fertilizer (Table 1, Table 3 and Table 5), which is partially supported by research on other crops [38,39]. Cheng et al. [40] showed that the complex interaction between biochar and N fertilizer could be explained by their various direct and indirect effects on the soil‒rhizosphere‒plant system. Similarly, in our study, this complex interaction was explained by their effect on the soil microenvironment and tomato plant growth. The PCA plot (Figure 1) clearly shows that different doses of biochar and N fertilizer had different effects on the soil microenvironment and tomato plants. N1C0, N1C30, N1C50, N1C70, N2C0, N3C0, N4C0 and other treatments were separated by PC1, which showed that all treatments with a 32% N reduction (N1) and all treatments without biochar (C0) were not beneficial to the soil‒plant system. All treatment combinations of N2, N3, or N4 with C30, C50, or C70 and soil‒plant parameters were on the right side of the PCA plot, which shows that reducing N fertilizer by 16% or 24% (N2 and N3) still improves the soil‒plant parameters, equal to or even better than normal N application (N4). The above results suggest that adding biochar to the soil can reduce the amount of applied N fertilizer without a loss in tomato yield, but too much reduction in N fertilizer will have an adverse effect. This is supported by research from Zhu et al. [41] that revealed that biochar increases the retention of soil inorganic N and promotes N turnover by affecting soil microbial properties, which means reducing the amount of N fertilizer and adding biochar will not affect the conversion rate of N. It is worth noting, however, that too much reduction in N fertilizer will lead to an insufficient N supply in tomatoes, which may be why a significant negative impact on tomatoes occurs in such cases.

Based on a comprehensive consideration of tomato yield, tomato VC, sugar‒acid ratio, and economic benefits, the combination of 35 t ha^−1^ biochar and 200 kg ha^−1^ N fertilizer, which are the average values of the aforementioned four optimal combinations of biochar and N fertilizer, is deemed to be the best application. This combination saves N fertilizer resources and is recommended as the optimal biochar and N fertilizer combination for local farmers. To the best of our knowledge, this study is the first to reveal the complex relationships between the combination of biochar and N fertilizer, tomato soil microenvironment, tomato plant growth, tomato quality, tomato production, and economic benefits under drip irrigation in northwest China. From the perspectives of soil microorganisms and tomato plant soil, our research provides new and important ideas about the combined effects of biochar addition and nitrogen fertilizer reduction on tomatoes (growth, quality, and yield) based on the silty clay loam soil of northwest China, which will be beneficial for both scientific and practical communities. The proposed biochar‒N application strategy and novel economic profit analysis on tomatoes also provide valuable information for the local farmers.

## 5. Conclusions

In summary, there is a significant combined effect of biochar and N on the soil microenvironment, tomato growth, tomato quality, and tomato yield. Biochar addition shows great potential for reducing the amount of N fertilizer and improving the PFP of N for tomatoes. However, adding too much biochar (70 t ha^−1^) and reducing the N fertilizer by too much (32%) does not lead to satisfactory results for the soil microenvironment, tomato growth, tomato quality, and tomato yield. Biochar addition could significantly enhance the soil microenvironment and tomato growth when the amount of N fertilizer applied was reduced by 16% or 24%, while the soil microenvironment and tomato growth were significantly negatively affected when the amount of N fertilizer was reduced by 32%. Adding biochar at 30 t ha^−1^ achieved the best tomato quality; the VC content was highest when the combined N fertilizer was reduced by 16%, and the sugar‒acid ratio was highest when the combined N fertilizer was reduced by 24%. Adding biochar at 50 t ha^−1^ and reducing the N fertilizer by 24% achieved the greatest tomato yield. In this case, biochar addition increased soil enzyme activity (urease, phosphatase, and catalase increased by 88.76% on average), microbial abundance (bacteria, fungi, and actinomycetes increased by 87.49% on average), leaf gas exchange parameters (gs, Pn, and Tr increased by 43.23% on average), and biomass (increased by 39.67%) compared with non-biochar treatment. Adding biochar at 30 t ha^−1^ and reducing N fertilizer by 16% achieved the greatest economic benefits. In this case, biochar addition increased soil enzyme activity (urease, phosphatase, and catalase activities increased by 39.48% on average), microbial abundance (bacteria, fungi, and actinomycetes increased by 59.4% on average), gas exchange parameters (gs, Pn, and Tr increased by 30% on average), and biomass (increased by 26.24%) compared with non-biochar treatment.

In conclusion, we recommend 35 t ha^−1^ biochar and 200 kg ha^−1^ N fertilizer as the best combination for local farmers based on a comprehensive consideration of tomato yield, tomato VC, sugar‒acid ratio, and economic benefits. The results of this research are of great significance for the scientific management, resource conservation, and environmental protection of tomato crops, as well as the development of sustainable agriculture.

## Figures and Tables

**Table 1:** Output of two-way ANOVA of urease, phosphatase, catalase, bacteria, fungi, and actinomycetes as affected by N (N1, N2, N3, and N4) and biochar (C0, C30, C50, and C70).

Factors	Urease	Phosphatase	Catalase	Bacteria	Fungi	Actinomycetes
N	***	***	***	***	***	***
C	***	***	***	***	***	***
N × C	***	**	**	***	***	*

*, **, and *** indicate significance level at *p* < 0.05, *p* < 0.01, and *p* < 0.001, respectively.

**Table 2:** Descriptive statistics of urease, phosphatase, catalase, bacteria, fungi, and actinomycetes, as affected by N (N1, N2, N3, and N4) and biochar (C0, C30, C50, and C70); each value is the mean value of three replicates ± standard error.

Variables	Nitrogen	Biochar			
		C0	C30	C50	C70
**Urease** **(mg g^−1^ 24 h^−1^)**	N1	1.48 ± 0.05 Cb	1.71 ± 0.09 Bc	1.78 ± 0.04 ABb	1.82 ± 0.03 Ab
	N2	1.78 ± 0.03 Ca	2.09 ± 0.06 Bb	2.33 ± 0.07 Aa	2.27 ± 0.06 Aa
	N3	1.81 ± 0.05 Ca	2.42 ± 0.06 Aa	2.33 ± 0.03 ABa	2.28 ± 0.02 Ba
	N4	1.84 ± 0.03 Ca	2.46 ± 0.04 ABa	2.38 ± 0.06 Aa	2.30 ± 0.07 Aa
**Phosphatase** **(μg g^−1^ 1 h^−1^)**	N1	8.67 ± 0.50 Cc	11.63 ± 0.47 Bb	12.17 ± 0.59 Ac	12.60 ± 0.40 Ac
	N2	10.52 ± 0.41 Cb	12.96 ± 1.39 Bb	16.36 ± 0.96 Aa	15.84 ± 1.40 Aa
	N3	12.37 ± 0.49 Da	15.07 ± 0.60 Aa	14.50 ± 0.50 Ab	14.13 ± 0.74 Cb
	N4	12.47 ± 0.63 Ba	15.05 ± 1.11 Aa	14.98 ± 0.95 Aab	13.54 ± 0.54 Abc
**Catalase** **(mg L^−1^)**	N1	0.62 ± 0.11 Bc	0.90 ± 0.06 Ac	0.96 ± 0.05 Ac	0.93 ± 0.06 Ac
	N2	0.72 ± 0.11 Dc	1.25 ± 0.06 Cb	2.01 ± 0.16 Aa	1.71 ± 0.06 Ba
	N3	0.92 ± 0.08 Bb	1.49 ± 0.09 Aa	1.43 ± 0.06 Ab	1.41 ± 0.07 Ab
	N4	1.12 ± 0.10 Ca	1.62 ± 0.21 Aa	1.39 ± 0.16 Bb	1.30 ± 0.14 Bb
**Bacteria** **(108 g^−1^)**	N1	2.24 ± 0.25 Ba	2.30 ± 0.44 ABb	2.66 ± 0.48 ABc	2.82 ± 0.40 Ab
	N2	2.81 ± 0.26 Da	3.67 ± 0.25 Ca	5.32 ± 0.36 Aa	4.33 ± 0.28 Ba
	N3	2.92 ± 0.17 Ca	4.11 ± 0.26 Ba	4.84 ± 0.36 Aab	3.81 ± 0.12 Ba
	N4	2.15 ± 0.42 Ca	4.06 ± 0.36 Ba	3.95 ± 0.31 Ab	3.95 ± 0.25 Ba
**Fungi** **(105 g^−1^)**	N1	1.22 ± 0.17 Bb	1.26 ± 0.15 ABc	1.48 ± 0.28 Ac	1.37 ± 0.16 ABc
	N2	1.71 ± 0.70 Ca	3.24 ± 0.30 Bb	3.17 ± 0.30 Aa	2.70 ± 0.29 Ba
	N3	2.02 ± 0.19 Bb	3.89 ± 0.23 Aa	3.48 ± 0.16 Aa	3.43 ± 0.35 Aa
	N4	2.71 ± 0.70 Cab	3.33 ± 0.27 Aa	3.05 ± 0.22 Ab	2.92 ± 0.14 Bb
**Actinomycetes** **(106 g^−1^)**	N1	1.69 ± 0.59 Bc	1.97 ± 0.30 ABb	2.00 ± 0.62 ABb	2.19 ± 0.56 Ab
	N2	2.50 ± 0.46 Ca	3.46 ± 0.45 BCa	4.70 ± 0.36 Aa	4.12 ± 0.30 Ba
	N3	2.75 ± 0.09 Cb	3.98 ± 0.20 ABa	4.59 ± 0.17 Aa	3.37 ± 0.26 Ba
	N4	2.30 ± 0.44 Ba	3.87 ± 0.25 Aa	4.16 ± 0.41 Aa	3.77 ± 0.23 Aa

Means followed by the same lowercase letter in each column and the uppercase letters in each row do not differ at the level of 0.05 probability by the Duncan’s test.

**Table 3:** Output of two-way ANOVA of leaf gas exchange parameters, tomato growth, and biomass as affected by N (N1, N2, N3, N4) and biochar (C0, C30, C50, C70).

Factors	Pn	gs	Tr	Plant Height	Stem Thickness	Biomass
N	***	***	***	***	***	***
C	***	***	***	***	**	***
N × C	***	*	*	**	*	***

*, **, and *** indicate significance levels at *p* < 0.05, *p* < 0.01, and *p* < 0.001, respectively.

**Table 4:** Descriptive statistics of leaf gas exchange parameters, tomato growth, and biomass as affected by N (N1, N2, N3, N4) and biochar (C0, C30, C50, C70); each value is the mean value of three replicates ± standard error.

Variables	Nitrogen	Biochar			
		C0	C30	C50	C70
**Pn (mmol m^−2^ s^−1^)**	N1	21.25 ± 1.07 Cc	26.32 ± 0.31 Bd	29.90 ± 0.59 Ad	28.90 ± 0.26 Ac
	N2	23.25 ± 1.07 Db	29.96 ± 0.72 Cb	32.77 ± 0.36 Bc	35.20 ± 1.15 Aa
	N3	24.08 ± 0.29 Dab	31.58 ± 0.34 Ca	35.86 ± 0.43 Aa	34.12 ± 0.45 Ba
	N4	25.08 ± 0.77 Ca	27.07 ± 0.59 Bc	33.61 ± 0.96 Ab	34.29 ± 0.88 Aa
**gs (mol m^−2^ s^−1^)**	N1	0.36 ± 0.03 Dc	0.43 ± 0.01 Cb	0.56 ± 0.03 Ab	0.50 ± 0.01 Bb
	N2	0.39 ± 0.04 Cbc	0.49 ± 0.04 Ba	0.62 ± 0.05 Aa	0.57 ± 0.02 Aa
	N3	0.41 ± 0.02 Cb	0.50 ± 0.01 Ba	0.60 ± 0.03 Aab	0.59 ± 0.02 Aa
	N4	0.46 ± 0.02 Ca	0.50 ± 0.04 BCa	0.61 ± 0.02 Aab	0.52 ± 0.04 Bb
**Tr (mmol m^−2^ s^−1^)**	N1	9.18 ± 0.14 Cb	11.28 ± 0.33 Bc	11.86 ± 0.38 ABc	12.23 ± 0.38 Ac
	N2	9.81 ± 0.49 Dab	11.63 ± 0.41 Cc	12.73 ± 0.59 Bb	13.71 ± 0.72 Ab
	N3	10.09 ± 0.08 Ca	13.73 ± 0.95 Ba	14.11 ± 0.39 Ba	15.10 ± 0.39 Aa
	N4	10.46 ± 0.34 Ca	12.50 ± 0.41 Bb	13.25 ± 0.32 Ab	14.07 ± 0.23 Ab
**Plant height (cm)**	N1	107.00 ± 1.50 Cd	122.17 ± 0.29 Bc	125.17 ± 0.29 Ad	126.33 ± 0.29 Ac
	N2	112.331.61 Cc	124.00 ± 0.50 Bb	131.87 ± 0.33 Ab	130.67 ± 1.26 Ab
	N3	114.55 ± 1.00 Cb	129.67 ± 0.29 Ba	133.33 ± 0.76 Aa	133.50 ± 0.87 Aa
	N4	115.33 ± 1.04 Ca	128.67 ± 1.04 Aa	130.47 ± 0.96 Bc	129.33 ± 1.04 ABb
**Stem thickness (cm)**	N1	12.15 ± 0.27 Bb	12.39 ± 0.18 Bc	12.66 ± 0.28 ABb	13.05 ± 0.18 Ab
	N2	13.38 ± 0.44 Ba	13.68 ± 0.45 Ab	14.25 ± 0.38 Aa	14.03 ± 0.53 Aa
	N3	13.45 ± 0.17 Aa	13.83 ± 0.19 Ab	13.92 ± 0.25 Aa	13.56 ± 0.14 Aa
	N4	13.82 ± 0.28 Ba	14.54 ± 0.20 Aa	14.14 ± 0.53 ABa	13.88 ± 0.16 Ba
**Biomass (g)**	N1	30.32 ± 0.37 Dd	40.36 ± 0.13 Ab	36.68 ± 0.30 Bc	31.94 ± 0.38 Cd
	N2	31.10 ± 0.46 Dc	40.67 ± 0.28 Bb	43.43 ± 0.07 Aa	39.71 ± 0.34 Ca
	N3	32.36 ± 0.49 Db	40.85 ± 0.85 Ba	42.34 ± 0.40 Ab	37.41 ± 0.54 Cc
	N4	33.80 ± 0.19 Da	40.08 ± 0.30 Bb	42.92 ± 0.39 Aab	38.35 ± 0.63 Cb

Means followed by the same lowercase letter in each column and the uppercase letter in each row do not differ at the level of 0.05 probability by the Duncan’s test.

**Table 5:** Output of two-way ANOVA of tomato yield, sugar‒acid ratio, VC, and PFP of N as affected by N (N1, N2, N3, N4) and biochar (C0, C30, C50, C70).

Factors	Yield	VC	Sugar‒Acid Ratio	PFP of N
N	***	***	***	***
C	***	***	***	***
N × C	***	***	ns	***

*, **, and *** indicate significance levels at *p* < 0.05, *p* < 0.01, and *p* < 0.001, respectively.

**Table 6:** Descriptive statistics of tomato yield, sugar‒acid ratio, VC, and PFP of N as affected by N (N1, N2, N3, N4) and biochar (C0, C30, C50, C70); each value is the mean value of three replicates ± standard error.

Variables	Nitrogen	Biochar			
		C0	C30	C50	C70
**Yield (t ha^−1^)**	N1	56.52 ± 0.33 Dd	85.91 ± 0.31 Cc	89.94 ± 0.29 Ab	88.06 ± 0.42 Bd
	N2	62.28 ± 0.18 Dc	90.78 ± 0.15 Cb	96.40 ± 0.16 Aa	94.21 ± 0.24 Ba
	N3	69.98 ± 0.22 Db	93.30 ± 0.22 Ca	96.30 ± 0.11 Aa	92.57 ± 0.20 Bb
	N4	72.72 ± 0.09 Da	93.27 ± 0.31 Ba	96.23 ± 0.30 Aa	91.90 ± 0.16 Cc
**VC (mg·100 g^−1^)**	N1	3.77 ± 0.08 Dd	4.28 ± 0.07 Bd	4.57 ± 0.08 Ad	4.26 ± 0.07 Cc
	N2	5.14 ± 0.14 Dc	6.57 ± 0.09 Bc	7.23 ± 0.08 Ab	5.70 ± 0.17 Cb
	N3	6.14 ± 0.07 Da	7.96 ± 0.10 Aa	7.75 ± 0.08 Ba	7.20 ± 0.19 Ca
	N4	5.42 ± 0.09 Db	6.63 ± 0.08 Ab	6.10 ± 0.08 Bc	5.85 ± 0.08 Cb
**Sugar‒acid ratio**	N1	5.16 ± 0.12	5.72 ± 0.37	5.56 ± 0.37	4.88 ± 0.08
	N2	9.27 ± 0.82	9.59 ± 0.56	9.54 ± 0.39	8.48 ± 0.58
	N3	8.77 ± 0.33	8.39 ± 0.77	8.86 ± 0.53	8.03 ± 0.27
	N4	6.90 ± 0.29	6.66 ± 0.17	6.70 ± 0.70	5.72 ± 0.52
**PFP of N (kg kg^−1^)**	N1	332.48 ± 1.94 Da	505.32± 1.81 Ca	529.07 ± 1.70 Aa	517.96 ± 2.45 Ba
	N2	327.79± 0.92 Db	477.78± 0.79 Cb	507.39 ± 0.82 Ab	495.85 ± 1.29 Bb
	N3	333.26 ± 1.05 Da	444.30 ± 1.04 Bc	458.59 ± 0.52 Ac	440.80 ± 0.93 Cc
	N4	290.89 ± 0.36 Dc	373.07± 1.24 Bd	384.92 ± 1.21 Ad	367.61 ± 0.62 Cd

Means followed by the same lowercase letter in each column and the uppercase letter in each row do not differ at the level of 0.05 probability by the Duncan’s test.

## Data Availability

Not applicable.

## Appendix A

**Table A1:** Cost‒benefit analysis of different biochar‒N applications treatments for tomatoes in a single greenhouse (10^3^ CNY).

Treatments	Yield (t/ha)	Input Cost					Income	Benefit
		Biochar	Fertilizer and Pesticide	Pipe System	Water and Electricity	Seedlings and Others		
N1C0	56.521	0.000	1.010	2.024	0.100	1.500	21.101	18.491
N1C30	85.904	4.000	1.010	2.024	0.100	1.600	32.071	25.361
N1C50	89.942	6.667	1.010	2.024	0.100	1.700	33.578	24.101
N1C70	88.053	9.333	1.010	2.024	0.100	1.800	32.873	20.630
N2C0	62.279	0.000	1.160	2.024	0.100	1.500	23.251	20.491
N2C30	90.779	4.000	1.160	2.024	0.100	1.600	33.891	27.031
N2C50	96.404	6.667	1.160	2.024	0.100	1.700	35.991	26.364
N2C70	94.212	9.333	1.160	2.024	0.100	1.800	35.172	22.779
N3C0	69.984	0.000	1.210	2.024	0.100	1.500	26.127	23.317
N3C30	93.303	4.000	1.210	2.024	0.100	1.600	34.833	27.923
N3C50	96.303	6.667	1.210	2.024	0.100	1.700	35.953	26.276
N3C70	92.567	9.333	1.210	2.024	0.100	1.800	34.558	22.115
N4C0	72.721	0.000	1.260	2.024	0.100	1.500	27.149	24.289
N4C30	93.268	4.000	1.260	2.024	0.100	1.600	34.820	27.860
N4C50	96.231	6.667	1.260	2.024	0.100	1.700	35.926	26.199
N4C70	91.904	9.333	1.260	2.024	0.100	1.800	34.311	21.818
